# Dealing With a Nightmare: A Case Report of Successful Percutaneous Treatment of an Embolized Occluder Device Complicating a Minimal-Fluoroscopy Patent Ductus Arteriosus Closure

**DOI:** 10.7759/cureus.61926

**Published:** 2024-06-07

**Authors:** Fransiska A Sihotang, Valerinna Putri

**Affiliations:** 1 Department of Cardiology and Vascular Medicine, Universitas Brawijaya, Saiful Anwar General Hospital, Malang, IDN

**Keywords:** patent ductus arteriosus, case report, snaring, device retrieval, device embolization, pda closure

## Abstract

﻿Patent ductus arteriosus (PDA) is one of the most prevalent acyanotic congenital heart diseases. Percutaneous closure of PDA has been the preferred treatment recommended by the guidelines due to relatively low complications and rapid patient recovery. However, device emboli remain the most frequent and disastrous complication, necessitating percutaneous or surgical treatment. We present a case of a large PDA closure in pulmonary arterial hypertension paediatric patients complicated with device emboli that was successfully retrieved using the snaring technique. Transcatheter retrieval, although technically challenging, is a feasible treatment and offers the advantage of avoiding the need for surgical intervention.

## Introduction

Patent ductus arteriosus (PDA) is an abnormal condition in which there is a persistent opening between the descending thoracic aorta, located below the left subclavian artery, and the pulmonary artery due to the abnormal persistence of the foetal ductus arteriosus that constitutes 5-10% of the total cases of congenital cardiac defects [1]. During foetal development, the ductus arteriosus enables the oxygenated blood from the placenta to bypass the foetal lungs. Following delivery, the lungs become inflated with air, resulting in a decrease in pulmonary vascular resistance (PVR) and blood flow from the right ventricle to the lungs for oxygenation. In healthy, full-term neonates, the ductus arteriosus constricts and functionally closes between 12 and 24 hours of age [1].

PDA can lead to many complications, including congestive heart failure, atrial arrhythmias, endocarditis, ductal aneurysms, pulmonary vascular disease, and Eisenmenger syndrome [1]. Transcatheter closure of PDA has emerged as the favoured therapeutic approach over surgery, as suggested by both the European Society of Cardiology (ESC) and the American Heart Association/American College of Cardiology (AHA/ACC) [2,3]. Haemodynamic consequences of the procedure are influenced by several factors, including the magnitude of the shunt, fluctuations in systemic and pulmonary artery pressure, vascular resistance, and the length and narrowest ductal diameter [4].

Fluoroscopy has remained the primary approach for guidance, despite the adjustments made to the occluder and device delivery systems over time. Due to advancements in technology, it is now possible to perform transcatheter PDA closure with minimal reliance on fluoroscopy. Following the pioneering work of Ewert et al., who achieved the first effective closure of atrial septal defect (ASD) using transoesophageal echocardiographic (TEE) guidance, numerous medical centres have investigated transcatheter procedures that require minimal or no use of fluoroscopy. This method reduces radiation exposure for both patients, especially children, and medical staff [5].

The occurrence of device embolization (DE) during percutaneous PDA closure is infrequent but has the potential to be life-threatening. The rate of DE ranges from 0% to 3.1% [6] and its clinical presentations might vary from incidental discovery during physical examination or imaging, to cardiogenic shock or cardiac arrest [7]. Embolization often occurs due to improper sizing or ineffective deployment of the device, even with careful planning and execution of the procedure. The management techniques for DE vary based on the specific characteristics of the embolized device, such as its type, size, and location, as well as the timing of diagnosis and the clinical profile of the patient [7]. Having a thorough knowledge of the instruments at hand and a clear awareness of the role and constraints of percutaneous retrieval procedures are crucial for effectively managing DE. We present a challenging case of a large PDA closure in pulmonary arterial hypertension paediatric patient using the minimal-fluoroscopy method complicated with device emboli that was successfully retrieved using the snaring technique.

## Case presentation

**Figure 1:**
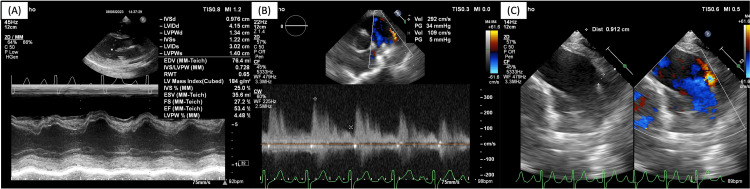
Pre-procedural transthoracic and transoesophageal echocardiography. Echocardiography showing (A) LV concentric hypertrophy with normal LV systolic function, (B) continuous left-to-right ductal flow, and (C) PDA diameter of 9 mm. LV, left ventricle; PDA, patent ductus arteriosus.

**Figure 2:**
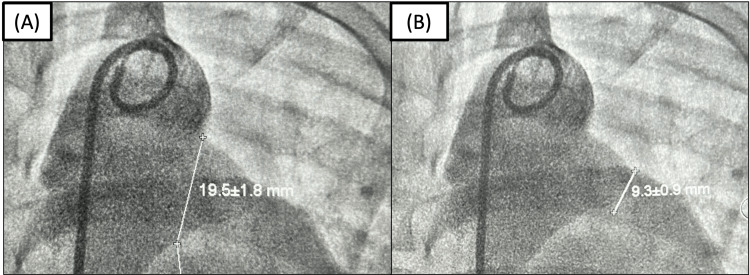
Pre-procedural right heart catheterization and aortography. Right catheterization result: flow ratio (Qp/Qs) = 3.3; pulmonary vascular resistance = 6.6 Woods unit (WU); PARi = 5.9 WU/m2; and PVR/SVR = 0.25 with mean pulmonary artery pressure of 61 mmHg (indicating pulmonary hypertension) and mean descending aorta pressure of 72 mmHg. Oxygen saturation of the pulmonary artery was 91% and descending aorta was 98%. Aortography measurement of the ductus revealed the diameter of the ampulla of 19.5 mm (A) and isthmus of 9.3 mm (B). Qp, pulmonary blood flow; Qs, systemic blood flow; PVR, pulmonary vascular resistance; SVR, systemic vascular resistance; PARi: pulmonary arterial resistance index.

A 12-year-old pulmonary hypertensive paediatric patient with Down syndrome presented with recurrent respiratory tract infection and growth retardation one year prior to diagnosis of PDA (Figures 1, 2). Right heart catheterization revealed mean pulmonary arterial pressure (mPAP) of 61 mmHg (Figure 2), with mPAP > 20 mmHg being the diagnostic criteria for pulmonary hypertension. The patient then underwent elective percutaneous PDA closure using a minimal-fluoroscopy technique. The patient was put under general anaesthesia and the initial measurement of PDA using transoesophageal echocardiography (TEE) was 9 mm isthmus and 22 mm ampulla. It was decided to use the MemoPart^TM^ PDA occluder No. 18/20 (Lepu Medical, Beijing, China) (twice the diameter of the isthmus) and antegrade transvenous PDA closure was performed. TEE confirmed proper device placement and the continuous murmur disappeared upon auscultation. The procedure concluded without any complications.

**Figure 3:**
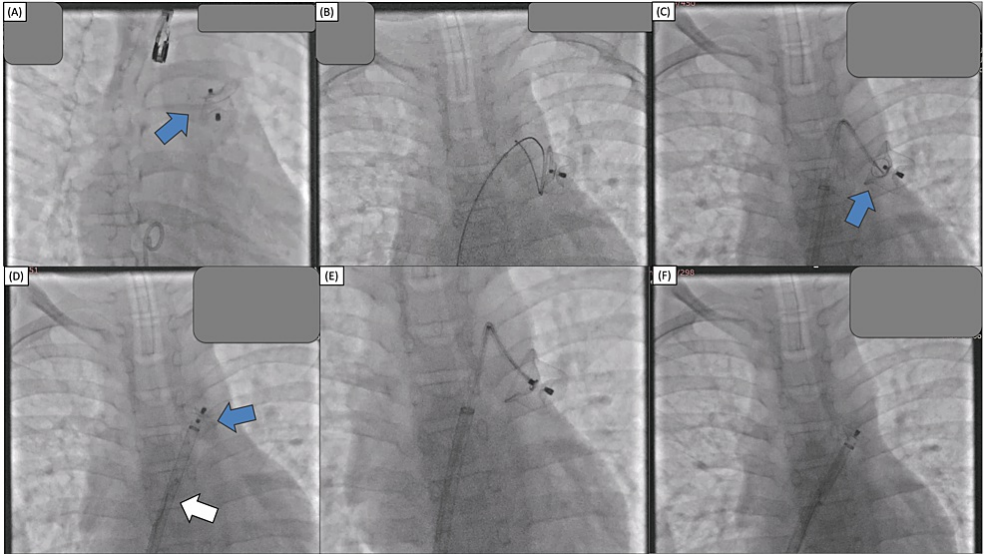
A: The PDA occluder (blue arrow) was in an awkward position (migrated to the LPA). B: The PDA occluder was rotated using a 6F guiding catheter and hydrophilic wire so that the aortic disc (retention disc) faces the pulmonary artery. C: A 14F delivery sheath was used to support the Judkins right 3.5 6F guiding catheter and the endovascular snare was advanced to capture the device hub. D: After several attempts, the snare was able to capture the hub; however, during retraction, the delivery sheath was detached from the snare (white arrow). E: Device snaring was attempted several times using different views and the hub was successfully captured. F: The device was successfully retrieved into the delivery sheath. LPA, left pulmonary artery; PDA, patent ductus arteriosus.

**Figure 4:**
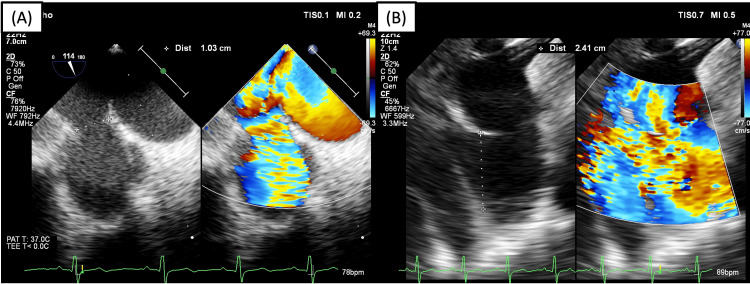
Re-evaluation of PDA size after device retrieval revealed (A) isthmus of 10 mm and (B) ampulla of 24 mm. We decided to use PDA occluder No. 20/22 (aortic waist ∅ 22 mm, pulmonic waist ∅ 20 mm, aortic disc ∅ 27 mm) with an antegrade approach. PDA, patent ductus arteriosus.

However, several hours later during a follow-up examination, the murmur had reappeared. Echocardiography revealed that the device migrated into the left pulmonary artery (Figure 3A). It was decided to perform immediate percutaneous device retrieval with the snaring technique through femoral vein access. First, the occluder was rotated using hydrophilic wire so the aortic disc faces the pulmonary artery (Figure 3B). A 6F guiding catheter was initially used to support the endovascular snare with unsuccessful result. Then, a 14F delivery sheath was advanced and parked at the main pulmonary artery followed by a 6F guiding catheter to provide more support and the endovascular snare was advanced towards the device (Figure 3C). We used endovascular snares with diameters of 10 mm and 30 mm interchangeably. After several attempts, the snare was finally able to capture the hub; however, during retrieval into the sheath, the device was detached from the snare (Figure 3D). After further attempts, the snare was able to capture the hub and the device was retrieved successfully into the delivery sheath (Figure 3F). The PDA size was re-evaluated using TEE, revealing a 10 mm isthmus and 22 mm ampulla (Figure 4). Subsequent closure was performed under combined fluoroscopy and TEE guidance, using the MemoPart^TM^ PDA occluder No. 20/22. Device placement was confirmed under fluoroscopy and TEE and there was no residual shunt.

During and after the procedure, the patient received an infusion of packed red cells (PRC) until the haemoglobin level was >10 grams (%). The patient showed no sign of infection, such as fever or leucocytosis. The patient was discharged three days after the procedure. The patient’s guardians were appreciative that the transcatheter device retrieval was successful, and that he did not have to undergo surgical intervention.

## Discussion

The ductus arteriosus is a vascular channel that connects the upper portion of the descending aorta to the pulmonary artery near the origin of the left branch of the pulmonary artery. In neonates, the closure of the ductus arteriosus occurs in more than 90% of instances within 48 hours, and in all cases by 96 hours after birth. Some individuals may continue to have PDA throughout later childhood or adulthood [8]. PDA is also found with increased frequency in several genetic syndromes, including Down syndrome (trisomy 21) [8].

We presented a case of a 12-year-old male paediatric patient with Down syndrome and growth retardation who was diagnosed with PDA in June 2023. Right heart catheterization showed pulmonary hypertension and the patient was planned for transcatheter PDA closure with zero or minimal fluoroscopy. Transcatheter closure has emerged as the primary method for closing most PDA cases due to its reduced complications and shorter hospitalization duration compared to surgery. Both the 2018 AHA/ACC and the 2020 ESC guidelines for the management of congenital heart disease recommend transcatheter closure as the preferred treatment for all PDA cases presenting with left ventricular (LV) volume overload, regardless of the presence of symptoms [2,3]. However, this treatment is not recommended for patients with Eisenmenger physiology, lower limb desaturation during exercise, pulmonary artery systolic pressure greater than two-thirds of systemic systolic pressure, or PVR greater than two-thirds of systemic vascular resistance in adults [2,3].

Our patient presented with a history of recurrent respiratory tract infection for one year prior to diagnosis of PDA and growth retardation. Pre-procedural echocardiography showed LV concentric hypertrophy with normal LV systolic function with continuous left-to-right ductal flow and a PDA diameter of 9 mm (Figure 1). Pre-procedural right heart catheterization and aortography showed pulmonary hypertension (mPAP = 61 mmHg) and PVR of 6.6 WU with a flow ratio of 3.3 (significant left-to-right shunt) and PVR/SVR of 0.25 (Figure 2). In this case, according to the 2020 ESC guidelines, the recommendation for PDA closure is class IC [2].

Transcatheter PDA closure, guided by fluoroscopy, has been the preferred method for the past 40 years [9]. This is because it offers the advantage of correctly localizing the wire and device, making it a convenient alternative. Following the initial achievement of the first echocardiography-guided balloon atrial septostomy conducted by Rashkind in 1966, numerous cardiologists have been exploring the use of echocardiography as a means of guidance for various procedures, including PDA closure, to reduce the amount of radiation that patients, especially paediatric patient, and medical professionals are exposed to.

Currently, there is a lack of definitive instructions on the appropriate method for determining the size of a ductal occluder. The majority of operators choose to insert devices that are at least 2 mm larger than the narrowest point of the duct [10]. Kanabar et al. (2020) suggested that in children, the size of the pulmonary end of the device should be at least 2 mm greater than the narrowest diameter of the PDA [11]. In adolescents and adults with severe pulmonary arterial hypertension, it should be twice the narrowest diameter [11]. In this case, we use a device two times the narrowest diameter of PDA due to pulmonary hypertension in this patient.

In a recent study conducted by Mumtaz et al. [12], it was discovered that device embolization occurred in 4% of transcatheter duct closures, in which surgery was required in 20% of these instances. Device embolization is more frequently observed in cases when there are larger ducts accompanied by increased pulmonary artery pressures [12]. Tubular ducts have a higher susceptibility to embolization when compared to typical conical ducts. Accurate echocardiographic measures, appropriate selection of occluder, meticulous technique, and extra caution in patients with elevated pulmonary artery pressures are essential to reduce the risk of embolization [12]. In this patient, it is probable that device emboli occurred due to insufficient imaging, resulting in an underestimation of the size of the duct. This was likely caused by limited visualization of the ductus and the adoption of an improperly sized device, which was too small for the anatomy encountered.

The retrieval strategies vary depending on the particular embolized device. It is recommended to initially attempt percutaneous retrieval as the primary approach for devices embolized to large arteries such as pulmonary arteries or aorta [7]. Initially, a basic snare (single-loop snares) can be employed to capture and recover the device. The snare can be delivered either directly through the retrieval sheath or through a curved coronary guiding catheter that is placed into the retrieval sheath. This curved catheter helps to improve the directionality and makes it easier to grasp the device. The location of snaring varies depending on the type of device and the presence or absence of end screw pins. It is important to be careful while attaching snare devices to the waist of occluders, as the snare may not be easily withdrawn afterwards [7]. Nitinol-based devices, like PDA occluders, can be folded and inserted into the sheath without compromising their structural integrity. The presence of "hookable" pins or hubs, such as the retention screw provided in the PDA occluder device, allows for the item to be retrieved [7]. Nevertheless, achieving a consistent pull after capturing the hub can frequently provide difficulties, as is the situation in our initial attempt.

Limitations of snaring include challenges in capturing the retention screw, potential device distortion during the snaring process, and the risk of damaging the vessel wall [13]. In our case, we used an antegrade approach with femoral vein access using an endovascular snare that was available at our centre. Because of the position of the device, it was first turned so that the aortic disc with a shallower hub was facing the pulmonary artery. After multiple attempts, snaring was successful after using a delivery sheath supporting guiding catheter harbouring the endovascular snare. The guiding catheter was advanced through the delivery sheath, which helped to effectively approximate the snare to the device, and the device was retrieved successfully. During the procedure, substantial bleeding was observed due to the large size of the delivery sheath, and the patient was given a blood transfusion during and after the procedure. Bleeding at the puncture site was managed with surgical haemostasis that was successfully removed the next day.

If the initial attempt to recapture is unsuccessful, it may be necessary to escalate to more advanced percutaneous retrieval techniques. These approaches involve the removal of an embolized device dislodged in a major blood vessel wall [7]. This is done using a bioptome or a simple catheter like a pigtail, to reposition the device. A further approach involves employing dual tool retrieval techniques, which entail the simultaneous use of two different instruments such as snares, snare and bioptome, or snare and forceps [7]. If after escalation it is still unsuccessful, then surgical retrieval should be performed.

A recent single-centre study conducted by Siagian et al. (2022) in Indonesia has demonstrated that the novel method of zero-fluoroscopy PDA closure was just as effective as the standard fluoroscopy-guided closure in terms of procedural success, procedure duration, and the frequency of adverse events [14]. Nevertheless, due to its novel nature, this approach is particularly reliant on the operator's skills and requires a proficient team to execute it well and prevent any procedural difficulties. Future endeavours should focus on establishing specific criteria for patient selection, taking into account the size of the PDA and related structures.

To minimize the occurrence of DE, it is crucial to engage in careful preparation, employ effective methods, and possess a sufficient understanding of the elements that contribute to a higher likelihood of DE in transcatheter PDA closure (Table 1) [15]. In our case, we conclude that a combination of operator-related factors such as unfamiliarity with the imaging technique, in this case, ductal measurement using TEE, and technique-related factor of selecting the appropriate device size contributed to the occurrence of DE. Once embolization occurs, however, effectively managing the situation relies on two crucial factors: (1) having access to percutaneous retrieval tools and a thorough understanding of their capabilities and limitations; and (2) following a systematic approach that combines percutaneous and surgical options, taking into consideration the operator's expertise and the patient's clinical condition [7]. Furthermore, a multidisciplinary collaboration is invaluable in achieving safe retrieval.

**Table 1:** Mechanisms for device embolization in transcatheter PDA closure. PDA: patent ductus arteriosus; "-": not available.

Operator-related factors	Technique-related factors	Patient-related factors
Pushing delivery wire or catheter forward after device release.	Underestimation of ductal dimension due to incomplete visualization of the ductus.	Vigorous activity results in a sudden increase in blood flow or intrathoracic pressure.
Prolonged time interval between device placement and release.	The implanted device is too small or does not match the shape of the ductal anatomy.	-
Inadvertent unscrewing of the device from the delivery wire.	Instrumentation of the ductus causes smooth muscle constriction leading to underestimation of ductal diameter.	-
Unfamiliarity with device sizing and placement guidelines.	Incorrect device orientation or shape.	-
-	Anterior tension on the device by delivery wire.	-

## Conclusions

Device embolization is one of the complications for percutaneous PDA closure, with rates varying between 0% and 3.1%. In our case, improper device selection due to the discrepancy of PDA size from the first and second TEE measurements resulted in device embolization. Transcatheter retrieval, although technically challenging, is a feasible treatment and offers the advantage of avoiding the need for surgical intervention. In addition, future efforts should prioritize the development of a standardized technique and more training in the use of zero- or minimal-fluoroscopy PDA closure.
